# Determinants of High Breastfeeding Self-Efficacy among Nursing Mothers in Najran, Saudi Arabia

**DOI:** 10.3390/nu15081919

**Published:** 2023-04-15

**Authors:** DaifAllah D. Al-Thubaity, Mohammed A. Alshahrani, Wafaa T. Elgzar, Heba A. Ibrahim

**Affiliations:** 1 Department of Maternity and Childhood Nursing, Nursing College, Najran University, Najran 66441, Saudi Arabia; 2 Department of Clinical Laboratory Sciences, Applied Medical Sciences College, Najran University, Najran 66441, Saudi Arabia; 3 Department of Obstetrics and Gynecologic Nursing, Nursing College, Damanhour University, Damanhour 22514, Egypt; 4 Department of Obstetrics and Woman Health Nursing, Benha University, Benha 13511, Egypt

**Keywords:** breastfeeding, self-efficacy, knowledge, attitude, Saudi Arabia

## Abstract

Many factors have been found to correlate with satisfactory Exclusive Breastfeeding (EBF) practices. The relationships between EBF practices and associated factors are complex and multidimensional; Breastfeeding Self-Efficacy (BSE) is the most important psychological factor that may help the mother to overcome any expected barriers. This study investigates the determinants of high breastfeeding self-efficacy among Saudi nursing mothers. Methods: This is a descriptive cross-sectional study investigating the determinant of BSE among 1577 nursing mothers in primary health centers in Najran City, Saudi Arabia. The study uses a cluster random sampling technique. Data collection was performed from June 2022 to January 2023 using a self-reported questionnaire that encompasses the Breastfeeding Self-Efficacy Scale—Short Form (BSES-SF), Gender Friendly Breastfeeding Knowledge Scale (GFBKS), Iowa Infant Feeding Attitude Scale (IIFAS), and a basic data questionnaire to assess women’s demographic factors and obstetric history. Results: The mean score for all BSES-SF items was between 3.23–3.41, the highest mean score was in mothers who felt comfortable breastfeeding with family members present (3.41 ± 1.06), and the lowest mean was in mothers who could breastfeed their baby without using formula as a supplement (3.23 ± 0.94). The overall BSE score was high among 67% of the study participants. Binary logistic regression showed that being a housewife, being highly educated, having breastfeeding experience, and being multiparous are positive predictors for high BSE (*p* ≤ 0.001). In addition, having adequate breastfeeding knowledge and positive breastfeeding attitudes were positively associated with higher BSE *(p* = 0.000). Conclusion: BSE can be predicted by modifiable predictors such as mothers’ education, working status, parity, breastfeeding experience, adequate breastfeeding knowledge, and positive attitudes toward breastfeeding. If such predictors are considered during breastfeeding-related educational interventions, it could lead to more effective and sustainable effects in community awareness regarding breastfeeding.

## 1. Introduction

Breastfeeding is the first and most important step toward healthy infants and communities. The World Health Organization recommends exclusive breastfeeding for the first 6 months of life, followed by the appropriate introduction of complementary foods with continued breastfeeding to two years and beyond [1]. Breastfeeding has evidence-based and well-known short- and long-term benefits for mothers and infants. Breast-milk composition is continually changed from one feed to another to meet the infant’s body requirements. Human milk contains easily digested proteins such as glycoproteins, enzymes, and endogenous peptides, which help enhance immunity, cognitive development, and gut maturation, promote healthy infant development, and support healthy microbial colonization [2]. Furthermore, the fat in human milk is an essential source of energy and a facilitator of cell functions [3]. Long-term benefits of human milk include decreased risk for asthma [4], gastrointestinal infections, and adult diabetes [5]. For mothers, breastfeeding helps rapid weight loss [6], delayed fertility [7], and decreases the risk of diabetes, cardiovascular diseases, elevated blood cholesterol, and some types of cancers [8].

Despite the well-known benefits of breastfeeding, its rate is still much lower than expected. The World Health Organization stated that less than half of infants worldwide have EBF during the first six months of life [1]. The range of EBF in Saudi Arabia is much lower than reported by WHO and varies by region. EBF ranged from 0.8 to 43.9% according to a systemic review that surveyed 17 studies [9]. Many factors were found to be correlated with satisfactory EBF practices. In addition, the relationships between EBF practices and associated factors are complex and multidimensional; BSE is the most important psychological factor that may help the mother overcome any expected barriers and may correlate to other EBF-associated factors [10].

The term ‘breastfeeding self-efficacy’ (BSE) was first coined by Dennis in 1999 and is defined as maternal self-confidence in her ability to practice and master breastfeeding satisfactorily [11]. The research on breastfeeding has shown improvement in breastfeeding practices due to high BSE and has identified it as an essential factor in maternal ideation behavior regarding EBF [12,13]. If a woman has high BSE, she will make greater effort and demonstrate increased persistence to improve her breastfeeding practice, including searching for breastfeeding knowledge and seeking support from the health team and significant others. She will also work to overcome perceived barriers or challenges restricting her ability to breastfeed her infant satisfactorily [10]. Women with low BSE are three times more prone to terminate breastfeeding early [14].

Likewise, conclusions of previous systematic reviews reported a positive predictive relation between BSE and breastfeeding initiation and continuation. In addition, interventions to improve BSE improved breastfeeding initiation and continuation at one and two months postpartum [15,16]. Although numerous studies have investigated the association between BSE and breastfeeding practices, the relationship between the two variables still needs to be completely understood. Some international studies investigated the predictors of BSE during the immediate postpartum period or pregnancy [17,18,19]. However, few studies have investigated BSE among Saudi women during pregnancy [20,21]. No published studies have investigated BSE during the first six months of an infant’s life, which is the most critical period in the breastfeeding span. Therefore, determining the BSE predictors during the first six months of life is crucial in anticipating breastfeeding initiation and continuity over time. Consequently, the current study investigates the determinants of high breastfeeding self-efficacy among Saudi nursing mothers.

## 2. Materials and Methods

### 2.1. Study Design and Setting

A descriptive cross-sectional study was conducted in four primary healthcare centers in Najran City, Saudi Arabia. Najran City is the largest city and capital of the Najran region. Najran region lies in southwestern the border with Yamen. The Najran population has numerous traditions and health beliefs related to breastfeeding, which may positively or negatively influence infant feeding practices. Najran City contains thirteen major primary health centers affiliated to the Ministry of Health [22]. 

### 2.2. Study Participants

The inclusion criteria were nursing mothers, with a child aged one day to less than six months, an absence of any breastfeeding contraindications such as HIV, or any condition that may hinder the continuity of EBF or affect milk production such as breast augmentation, lift, or reduction, or nipple surgery, aged 18 years or over, literate, and willing to participate in the study. 

#### 2.2.1. Sample Size Determinations and Sampling Procedures

The sample size was calculated based on the following formula:

$$n=\frac{(df)(t^{2})P\times Q}{d^{2}}=\frac{\left(4\right){\left(1.96\right)}^{2}0.5\times 0.5}{{\left(0.05\right)}^{2}}=1536$$

where *n* = sample size, *df* = design effect of cluster sampling, *t* = the parameter related to the precision of obtaining the largest sample size (1.96 for an error risk of 5%) where the normal curve cuts off an area at the tails (the desired confidence level is 95%), *p* = expected prevalence of high BSE, *q* = 1 − *p* the expected proportion of moderate or low BSE, *d* = maximum tolerable error (the desired level of precision). The sample size was 1689, after adding 10% to compensate the anticipated sample loss or non-response rate. 

Najran City contains thirteen primary health centers; the researchers randomly selected 30% of the primary health centers (4 centers). The total sample size of 1689 was divided equally among the four primary health centers (422 participants from each center). In each center, all clinics dealing with children aged from 1 day to less than six months were included in the study. A proportional sample based on the follow-up rate was selected in each clinic, using the convenience sampling technique. The clinic nurse acted as a facilitator during the sampling process. Eight data collectors were distributed in the four centers (two in each center). Data collection took place from June 2022 to January 2023, three days per week from 9 a.m. until 2 p.m. 

**Figure 1:**
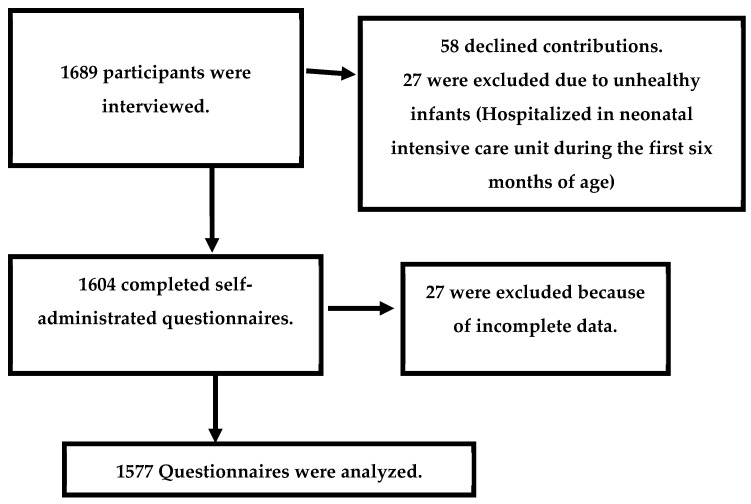
Participant flow chart.

The study participants were allocated according to Figure 1.

#### 2.2.2. Study Variables

The study’s dependent variable was BSE, and the independent variables were women’s demographic characteristics, obstetric history, previous breastfeeding experience, breastfeeding knowledge, and attitude.

### 2.3. Study Measurement Tools 

BSE was evaluated using BSES-SF, developed by Dennis to assess breastfeeding confidence among puerperal women. The BSES-SF was rated on a 5-point Likert scale ranging from 1 (not at all confident) to 5 (always confident). The total scale score ranged from 14–70, classified as low self-efficacy from 14–42 and high self-efficacy from 43–70. Based on the study by Amini et al., the BSES-SF had good internal consistency (r = 0.910) [23]. The psychometric analysis of the BSES-SF Arabic version was tested in the United Arab Emirates and revealed a highly reliability measure (α = 0.95) [24].

The maternal breastfeeding knowledge was evaluated using the Gender Friendly Breastfeeding Knowledge Scale (GFBKS), created by Gupta et al. It comprised 18 statements rated on a 5-point Likert scale (scored as 1 = false, 2 = maybe false, 3 = don’t know, 4 = maybe true, 5 = true). The GFBKS content validity was more than 0.80. The total scale score ranged from 18 to 90; the score for inadequate knowledge was 18–54, and adequate knowledge was from 55–90 [25]. The GFBKS Arabic version was validated by Tamim et al. and showed acceptable internal consistency (0.652) [26].

The Iowa Infant Feeding Attitude Scale (IIFAS) was utilized to evaluate women’s attitudes to breastfeeding. IIFAS was created by De la Mora et al., and contains 17 items ranked on a 5-point Likert scale, from 1 (strongly disagree) to 5 (strongly agree). Overall IIFAS scores ranged from 17 to 85; each woman was considered to have a negative (17–51) or positive attitude (52–85) based on her score. IIFAS items’ internal consistency ranged from 0.85 to 0.86 [27]. Charafeddine et al. assessed the psychometric properties of the IIFAS Arabic version with a sample of Lebanese women and found acceptable internal consistency (0.640) [28]. The scale was also validated in a study on Saudi pregnant women; the Cronbach alpha was 0.595 [29].

The researchers developed a basic data questionnaire to evaluate the women’s demographic characteristics and obstetric history. Demographic data include age, occupation, residency, monthly income, and women’s and their husbands’ education. Obstetric history included gravidity, parity, number of living children, duration of pregnancy, complications during the most recent pregnancy and delivery, mode of delivery for the most recent child, and breastfeeding experience. 

### 2.4. Data Collection Procedures and Technique

Data collection w from June 2022 to January 2023, three days weekly from 9 am until 2 pm. The data collection team comprised eight trained data collectors with previous experience in data collection. The data collectors were present in the waiting room in each clinic to identify the eligible participants based on the inclusion criteria. For each eligible participant, the data collector explained the study purpose and participant’s role and obtained informed consent. A self-administered questionnaire was completed in the presence of the data collector. The data collector’s role was to answer queries, clarify any concerns, and ensure data completeness in the questionnaire. 

### 2.5. Data Quality Control

The data collectors have bachelor’s degrees in nursing and previous data collection experience. Before data collection, the research team held two training sessions for the data collectors. The first session explained the research proposal, procedure, and ethics. At the end of the session, a copy of the data collection instrument was given to the data collectors to read before the next meeting. In the next session, one of the researchers provided a complete explanation of the questionnaire and clarified any queries. After data collection and during data entry, 27 questionnaires containing missing data were excluded from the analysis. 

### 2.6. Ethical Approval

After research proposal approval from the deanship of scientific research, the proposal and data collection tools were assessed and approved by the Najran health affairs ethical committee; ethical approval 2023-02 E. Permission to start data collection was also obtained from the MCH administration. Informed consent was taken from each mother before starting data collection. Participant anonymity was applied, and data were utilized only for research purposes. The participants were informed about their rights to decline participation without any penalties or consequences for the care provided. 

### 2.7. Data Analysis 

Data analysis was performed using Statistical IBM, version 23 ‘(IBM Corp., Armonk, NY, USA)’. Data were described using numbers, percentages, means, and standard deviation. The total BSE, knowledge, and attitude scores were obtained by summing items. Chi-square (X^2^) and Fisher exact tests (FET) were used to test group differences. Predictors of high BSE were examined through binary logistic regression. All the independent variables were categorical, and the first category was considered a reference. All factors were analyzed for multicollinearity in the regression model. The final model was checked with the Cox and Snell R-square goodness-of-fit test. Results were judged statistically significant at *p* < 0.05.

## 3. Results 

The frequency distribution of nursing mothers by demographic characteristics and overall breastfeeding self-efficacy scores are illustrated in Table 1. More than three-quarters (78.8%) of the nursing mothers were between the ages of 20–35, and the majority (93.6%) were urban residents. More than half (55.9%) were housewives, and 51.4% reported insufficient family income. Regarding mothers’ and their husbands’ education, 55.0% and 66.6% had a university education, respectively. BSE was significantly high among housewives and university-educated mothers (*p* = 0.000).

Approximately one-third (32.1%) of the nursing mothers breastfed exclusively, and 32.3% were primiparas. Around two-thirds (66.9%) delivered vaginally, and 19.7% reported complications during the most recent delivery. Concerning the participants’ overall knowledge and attitudes toward BF, more than half (60.2%) had adequate breastfeeding knowledge and 56.9% had a positive breastfeeding attitude. Moreover, exclusive breastfeeding, parity, adequate knowledge, and positive attitude were associated with higher BSE (*p* = 0.000) (Table 2).

The mean scores and standard deviation of BSES-SF items among nursing mothers are represented in Table 3. The overall mean BSES-SF score was 51.31 ± 10.79, with the mean score ranging from 3.23–3.41 for all BSES-SF items. As shown in the table, the highest mean score was for mothers who felt comfortable breastfeeding with family members present (3.41 ± 1.06), and the lowest mean was in mothers who could breastfeed their baby without using formula as a supplement (3.23 ± 0.94). 

Table 4 presents the binary logistic regression analysis of high BSE predictors. Occupational status was significantly associated with high BSE. Housewives had a 1.6 times higher probability of having high BSE (AOR 1.686; 95% CI 1.23–2.30, *p*  =  0.001) when taking employed mothers as a reference. Educational level was also a significant predictor of high BSE. Higher odds ratios were found in mothers who had received university (OR 69.474; 95% CI 39.52–122.11, *p* = 0.000) and secondary education (OR 45.140; 95% CI 27.95–72.88, *p*  = 0.000) compared with mothers who only could read and write. Furthermore, breastfeeding experience was significantly associated with high BSE. A mother who breastfed exclusively had a 5.9 times higher probability of having high BSE (OR 5.949; 95% CI 1.35–26.10, *p*  =  0.000) compared with non-exclusively breastfeeding mothers. In addition, multiparous mothers had a 3.1 times higher probability of having high BSE (OR 3.170; 95% CI 1.96–5.120, *p*  =  0.000) when compared with their primiparous counterparts. Finally, having adequate breastfeeding knowledge and positive attitudes were positively associated with higher BSE (OR 2.769; 95% CI 1.88–4.064, *p*  =  0.000; and OR 4.803; 95% CI 2.60–8.85, *p*  =  0.000, respectively).

## 4. Discussion

The present study findings reveal that approximately two-thirds of the nursing mothers had a high BSE. The significant determinants of high BSE included high educational level, being a housewife, having adequate knowledge, and having positive attitudes regarding breastfeeding. Multiparous mothers and mothers who had previously exclusively breastfed their babies also had a high BSE. The current study findings can help health professionals and decision-makers to design and implement supportive interventions to improve maternal BSE. Increasing BSE will facilitate and accelerate the improvement of breastfeeding practices for the benefit of nursing mothers and their infants.

In the present study, the overall mean BSES-SF scores of 1577 Saudi nursing mothers were moderate, with a mean score of 51.31 ± 10.79 out of 70. The results indicate that Saudi nursing mothers are confident in breastfeeding their infants. Along the same lines, a cross-sectional study was conducted by Khresheh and Ahmad to evaluate the associated demographic variables of BSE among pregnant participants in Saudi Arabia. Their results indicated moderate to relatively high averages for a prenatal BSE scale of 70 out of 100. They also added that BSE is a significant variable affecting breastfeeding practice [21].

Similar mean scores were also reported among other participants in international studies using the same instrument: 50.80 ± 8.91 in Iran [23] and 49.7 in Cyprus [30]. In contrast, the BSE mean was much higher in Turkey (55.13 ± 8.39) than was documented in the current study [31], while a lower BSE mean (47.3 ± 10.50) was reported in China [32]. Among the aforementioned studies, the research by Mercan et al. [31] evaluated BSE in the first 42 days of the postpartum period. The research by Ip et al. [32] conducted a longitudinal cohort study and followed up to six months after delivery. The differences between the current study and the aforementioned studies may be attributed to the differences between the studies’ designs and the data collection period.

This study revealed that a higher educational level significantly affected the BSE scale score. There is no doubt that educational level greatly affects breastfeeding knowledge and awareness. A high educational level may enhance BSE, empowering the mother in terms of health-seeking behavior and health education. Prior studies emphasized the important role of a high educational level in raising women’s BSE and consequently enhancing successful breastfeeding practices [33,34]. In addition, cross-sectional surveys conducted in Taiwan suggested that educational level was positively associated with an increased likelihood of BSE, particularly among university-educated mothers [35]. 

According to Bandura’s self-efficacy theory, if a person has good experience, their expectations will be higher [36]. In the current study, multiparous mothers with experience of exclusive breastfeeding had a higher probability of high BSE. According to Elgzar et al., previous experience of motherhood may initiate internal confidence in infant care and consequently shape maternal ideation behaviors regarding breastfeeding [37]. In several studies, higher BSE was related to previous positive breastfeeding experiences [37,38,39]. In addition, multi-parity was associated with successful breastfeeding among Saudi mothers in Al Hassa City [40]. The mediating role of BSE in successfully initiating and continuing breastfeeding practices has been documented in national and international studies [21,41,42,43]. 

Another important finding was the significant association between employment situation and the BSE score. Our findings revealed that housewives were 1.6 times more likely to have a high BSE compared with working mothers. This result was similar to other studies where working mothers outside their homes had low BSE and high barriers to optimal breastfeeding practices [44,45]. In addition, a recent qualitative study in Saudi Arabia showed that workplace policies and short periods of maternity leave (45–70 days) were connected with working mothers’ early introduction of supplementary feeding or early weaning [46]. Therefore, it is necessary to establish supportive rules to ensure that breastfeeding breaks for working nursing mothers are available in the workplace. An Ethiopian study reported that increasing the period of breastfeeding leave from work and establishing childcare centers near workplaces significantly improved breastfeeding practices among employed mothers [47].

The present study found that adequate breastfeeding knowledge and positive attitudes predict higher BSE. At a national level, a recent Saudi study showed that mothers with gestational diabetes mellitus who had good breastfeeding knowledge were more likely to have higher BSE [33]. The significant role of knowledge in the current study indicates the importance of promoting strategies to improve nursing mothers’ breastfeeding awareness and self-efficacy. Previous international studies have reported the benefits of educational interventions for enhancing mothers’ BSE and breastfeeding rates [13,48]. Consistent with prior studies [18,49], positive attitudes toward breastfeeding among nursing mothers were also significantly linked with higher BSE in the present study. Attitude toward breastfeeding has also been recognized as a predictor of breastfeeding behavior among women in Western Saudi Arabia [50]. Thus, educating pregnant and postpartum mothers to improve their knowledge and develop positive attitudes toward breastfeeding may enhance BSE and improve breastfeeding practices among women in Saudi Arabia.

### Strengths and Limitations

Our study has numerous strengths. A large sample size acquired using a random cluster sampling technique provides sufficient power to analyze the role of various predictors of BSE. Furthermore, this is the first study in Saudi Arabia to investigate the determinants of BSE in nursing mothers of babies under six months of age. Some limitations are also worthy to be mentioned. The current data were collected using a self-reported questionnaire, which may be susceptible to recall bias. In addition, it was not effectively possible to apply a random sampling technique to select the participants from each primary health center; therefore, we used a convenience sample. 

## 5. Conclusions

The current study found that mothers’ education, working status, parity, breastfeeding experience, adequate breastfeeding knowledge, and positive attitudes were predictors of BSE. If such predictors are considered during breastfeeding-related educational interventions, it could lead to more effective and sustainable effects in community awareness regarding breastfeeding. Despite the great efforts made by the Saudi Ministry of Health to raise community awareness regarding breastfeeding, multifaceted breastfeeding educational interventions, counseling, and support are needed to improve mothers’ BSE and thereby enhance proper breastfeeding practices.

## Figures and Tables

**Table 1:** Frequency distribution of the nursing mothers by demographic characteristics and overall breastfeeding self-efficacy score.

Variables	Total Sample N = 1577		Breastfeeding Self-Efficacy				X^2^/FET	*p*
			Low n = (520)		High n = (1057)			
	n	%	n	%	n	%		
**Age** (years)								
− <20	79	5.0	34	6.5	45	4.3	3.835	0.147
− 20–35	1242	78.8	404	77.7	838	79.3		
− ≥36	256	16.2	82	15.8	174	16.5		
**Residence**							0.081	0.775
− Rural	101	6.4	32	6.2	69	6.5		
− Urban	1476	93.6	488	93.8	988	93.5		
**Occupational status**							51.462	0.000 **
− Employee	696	44.1	296	56.9	400	37.8		
− Housewife	881	55.9	224	43.1	657	62.2		
**Education**							542.085	0.000 **
− University education	867	55.0	198	38.1	669	63.3		
− Secondary education	375	23.8	51	9.8	324	30.7		
− Read and write	335	21.2	271	52.1	64	6.1		
**Husband education**							3.267	0.195
− University education	1050	66.6	353	67.9	697	65.9		
− Secondary education	487	30.9	159	30.6	328	31.0		
− Read and write	40	2.5	8	1.5	32	3.0		
**Monthly income**							5.445	0.066
− Sufficient and save	197	12.5	65	12.5	132	12.5		
− Sufficient	810	51.4	247	47.5	563	53.3		
− Insufficient	570	36.1	208	40.0	362	34.2		

X^2^: Chi-square FET Fisher exact tests ** significant at *p* < 0.001.

**Table 2:** Frequency distribution of nursing mothers by breastfeeding experience, obstetric history, overall breastfeeding knowledge, attitude, and self-efficacy scores.

Variables	Total Sample N = 1577		Breastfeeding Self-Efficacy				X^2^/FET	*p*
			Low n = (520)		High n = (1057)			
	n	%	n	%	n	%		
**breastfeeding experience**							15.085	0.000 **
- Exclusive	506	32.1	133	25.6	373	35.3		
- Nonexclusive	1071	67.9	387	74.4	684	64.7		
**Parity**							64.942	0.000 **
- Primiparous	510	32.3	228	43.8	282	26.7		
- Multiparous	1067	67.7	292	56.2	775	73.3		
**Mode of delivery**							0.804	0.370
- Vaginal delivery	1055	66.9	340	65.4	715	67.6		
- Cesarean section	522	33.1	180	34.6	342	32.4		
**Complications during the last delivery**							2.017	0.165
- No	1266	80.3	428	82.3	838	79.3		
- Yes	311	19.7	92	17.7	219	20.7		
**Duration of pregnancy for the last child**							2.469	0.116
- Full-term	1430	90.7	463	89.0	967	91.5		
- Preterm	147	9.3	57	11.0	90	8.5		
**Overall knowledge**							124.376	0.000 **
- Inadequate (18–54)	628	39.8	309	59.4	319	30.2		
- Adequate (55–90)	949	60.2	211	40.6	738	69.8		
**Overall attitude**							96.397	0.000 **
- Negative (17–51)	680	43.1	315	60.6	365	34.5		
- Positive (52–85)	897	56.9	205	39.4	692	65.5		

X^2^: Chi-square FET Fisher exact tests ** significant at *p* < 0.001.

**Table 3:** Mean scores and standard deviation of BSES-SF items among nursing mothers.

BSES-SF Items	Mean	SD
Determine that my baby is getting enough milk	3.32	0.95
2. Successfully cope with breastfeeding as I have with other challenging tasks	3.37	0.90
3. Breastfeed my baby without using formula as a supplement	3.23	0.94
4. Ensure that my baby is properly latched on for the whole feeding	3.33	0.99
5. Manage the breastfeeding situation to my satisfaction	3.30	1.05
6. Manage to breastfeed even if my baby is crying	3.25	0.97
7. Keep wanting to breastfeed	3.38	1.07
8. Comfortably breastfeed with my family members present	3.41	1.06
9. Be satisfied with my breastfeeding experience	3.28	1.11
10. Deal with the fact that breastfeeding can be time-consuming	3.40	1.05
11. Finish feeding my baby on one breast before switching to the other breast	3.31	1.09
12. Continue to breastfeed my baby for every feeding	3.24	1.09
13. Manage to keep up with my baby’s breastfeeding demands	3.26	1.07
14. Tell when my baby is finished breastfeeding	3.24	1.11
**Overall mean of the BSES-SF score**	51.31	10.79

**Table 4:** Binary logistic regression analysis of high BSE predictors.

Predictors	High Breastfeeding Self-Efficacy	
	AOR (95% CI)	*p*
**Age** (years)		0.167
− <20	Ref	
− 20–35	0.630 (0.18–2.12)	0.456
− ≥36	0.591 (0.34–1.02)	0.059
**Residence**		
− Rural	Ref	
− Urban	0.844 (0.48–1.48)	0.556
**Occupational status**		
− Employee	Ref	
− Housewife	1.686 (1.23–2.30)	0.001 *
**Education**		0.000 **
− Read and write	Ref	
− Secondary education	45.140 (27.95–72.88)	0.000 **
− University education	69.474 (39.52–122.11)	0.000 **
**Husband education**		0.223
− Read and write	Ref	
− Secondary education	1.180 (0.32–4.30)	0.803
− University education	0.857 (0.24–3.01)	0.810
**Monthly income**		0.055
− Insufficient	Ref	
− Sufficient	0.965 (0.69–1.33)	0.830
− Sufficient and save	1.909 (1.09–3.33)	0.033 *
**breastfeeding experience**		
- Nonexclusive	Ref	
- Exclusive	5.949 (1.35–26.10)	0.000 *
**Parity**		
- Primiparous	Ref	
- Multiparous	3.170 (1.96–5.120)	0.000 **
**Mode of delivery**		
- Vaginal delivery	Ref	
- Cesarean section	1.090 (0.77–1.532)	0.622
**Complications during the last delivery**		
- No	Ref	
- Yes	0.612 (0.34–1.10)	0.102
**Duration of pregnancy for the last child**		
- Full-term		
- Preterm	1.095 (0.28–4.160)	0.894
**Overall knowledge**		
- Inadequate (18–54)	Ref	
- Adequate (55–90)	2.769 (1.88–4.064)	0.000 **
**Overall attitude**		
- Negative (17–51)	Ref	
- Positive (52–85)	4.803 (2.60–8.85)	0.000 **
−2 Log likelihood (1263.883)	Cox and Snell R Square (0.374)	Nagelkerke R Square (0.519)

AOR: Adjusted odds ratio, CI: confidence interval, * significant at *p* < 0.05, ** significant at *p* < 0.001.

## Data Availability

Data will be made available by the corresponding author upon reasonable request.

## Abbreviations

AOR	Adjusted odds ratio
EBF	Exclusive breastfeeding
GFBKS	Gender-Friendly Breastfeeding Knowledge Scale
BSES-SF	Breastfeeding Self-Efficacy Scale—Short Form
BSE	Breastfeeding self-efficacy
IIFAS	Iowa Infant Feeding Attitude Scale

## References

[1] World Health Organization WHO Breastfeeding [Internet]. https://www.who.int/health-topics/breastfeeding#tab=tab_1.

[2] Zhu J., Dingess K.A. (2019). The Functional Power of the Human Milk Proteome. Nutrients.

[3] Wesolowska A., Brys J., Barbarska O., Strom K., Szymanska-Majchrzak J., Karzel K., Pawlikowska E., Zielinska M.A., Hamulka J., Oledzka G. (2019). Lipid Profile, Lipase Bioactivity, and Lipophilic Antioxidant Content in High Pressure Processed Donor Human Milk. Nutrients.

[4] Oddy W.H. (2017). Breastfeeding, Childhood Asthma, and Allergic Disease. Ann. Nutr. Metab..

[5] Nuzzi G., Trambusti I., DICicco M.E., Peroni D.G. (2021). Breast milk: More than just nutrition!. Minerva Pediatr..

[6] da Silva M.d.C., Oliveira Assis A.M., Pinheiro S.M., de Oliveira L.P., da Cruz T.R. (2015). Breastfeeding and maternal weight changes during 24 months postpartum: A cohort study. Matern. Child Nutr..

[7] Calik-Ksepka A., Stradczuk M., Czarnecka K., Grymowicz M., Smolarczyk R. (2022). Lactational Amenorrhea: Neuroendocrine Pathways Controlling Fertility and Bone Turnover. Int. J. Mol. Sci..

[8] Binns C., Lee M., Low W.Y. (2016). The Long-Term Public Health Benefits of Breastfeeding. Asia-Pac. J. Public Health.

[9] Al Juaid D.A., Binns C.W., Giglia R.C. (2014). Breastfeeding in Saudi Arabia: A review. Int. Breastfeed J..

[10] Rosenblad A.K., Funkquist E.L. (2022). Self-efficacy in breastfeeding predicts how mothers perceive their preterm infant’s state-regulation. Int. Breastfeed J..

[11] Dennis C.L. (1999). Theoretical underpinnings of breastfeeding confidence: A self-efficacy framework. J. Hum. Lact..

[12] Anaba U.C., Johansson E.W., Abegunde D., Adoyi G., Umar-Farouk O., Abdu-Aguye S., Hewett P.C., Hutchinson P.L. (2022). The role of maternal ideations on breastfeeding practices in northwestern Nigeria: A cross-section study. Int. Breastfeed. J..

[13] You H., Lei A., Xiang J., Wang Y., Luo B., Hu J. (2020). Effects of breastfeeding education based on the self-efficacy theory on women with gestational diabetes mellitus: A CONSORT-compliant randomized controlled trial. Medicine.

[14] Vieira E.S., Caldeira N.T., Eugênio D.S., Lucca MM D., Silva I.A. (2018). Breastfeeding self-efficacy and postpartum depression: A cohort study. Rev. Lat.-Am. Enferm..

[15] Galipeau R., Baillot A., Trottier A., Lemire L. (2018). Effectiveness of interventions on breastfeeding self-efficacy and perceived insufficient milk supply: A systematic review and meta-analysis. Matern. Child Nutr..

[16] Maleki A., Faghihzadeh E., Youseflu S. (2021). The Effect of Educational Intervention on Improvement of Breastfeeding Self-Efficacy: A Systematic Review and Meta-Analysis. Obstet. Gynecol. Int..

[17] Melo L.C.O., Bonelli M.C.P., Lima R.V.A., Gomes-Sponholz F.A., Monteiro J.C.D.S. (2021). Anxiety and its influence on maternal breastfeeding self-efficacy. Rev. Lat.-Am. Enferm..

[18] Li L., Wu Y., Wang Q., Du Y., Friesen D., Guo Y., Dill S.E., Medina A., Rozelle S., Zhou H. (2022). Determinants of breastfeeding self-efficacy among postpartum women in rural China: A cross-sectional study. PLoS ONE.

[19] Piro S.S., Ahmed H.M. (2020). Impacts of antenatal nursing interventions on mothers’ breastfeeding self-efficacy: An experimental study. BMC Pregnancy Childbirth.

[20] Mosher C., Sarkar A., Hashem A.A., Hamadah R.E., Alhoulan A., AlMakadma Y.A., Khan T.A., Al-Hamdani A.K., Senok A. (2016). Self-reported breast feeding practices and the Baby Friendly Hospital Initiative in Riyadh, Saudi Arabia: Prospective cohort study. BMJ Open.

[21] Khresheh R.M., Ahmad N.M. (2018). Breastfeeding self efficacy among pregnant women in Saudi Arabia. Saudi Med. J..

[22] (2018). Ministry of Municipal and Rural Affairs and United Nations Human Settlements Programme. https://unhabitat.org/sites/default/files/2020/04/cpi_profile_for_najran_2019.pdf.

[23] Amini P., Omani-Samani R., Sepidarkish M., Almasi-Hashiani A., Hosseini M., Maroufizadeh S. (2019). The Breastfeeding Self-Efficacy Scale-Short Form (BSES-SF): A validation study in Iranian mothers. BMC Res. Notes.

[24] Radwan H., Fakhry R., Boateng G.O., Metheny N., Bani Issa W., Faris M.E., Obaid R.S., Al Marzooqi S., Al Ghazal H., Dennis C.L. (2023). Translation and Psychometric Evaluation of the Arabic Version of the Breastfeeding Self-Efficacy Scale-Short Form among Women in the United Arab Emirates. J. Hum. Lact. Off. J. Int. Lact. Consult. Assoc..

[25] Gupta A., Aravindakshan R., Sathiyanarayanan S., Naidu N.K., Santhoshi K.N.K.S., Kakkar R. (2022). Validation of Gender Friendly Breastfeeding Knowledge scale among young adults. J. Prev. Med. Hyg..

[26] Tamim H., Ghandour L.A., Shamsedine L., Charafeddine L., Nasser F., Khalil Y., Nabulsi M. (2016). Adaptation and Validation of the Arabic Version of the Infant Breastfeeding Knowledge Questionnaire among Lebanese Women. J. Hum. Lact..

[27] De la Mora A., Russell D.W., Dungy C.I., Losch M., Dusdieker L. (1999). The Iowa Infant Feeding Attitude Scale: Analysis of reli-ability and validity. J. Appl. Soc. Psychol..

[28] Charafeddine L., Tamim H., Soubra M., de la Mora A., Nabulsi M. (2016). Research and Advocacy Breastfeeding Team. Validation of the Arabic Version of the Iowa Infant Feeding Attitude Scale among Lebanese Women. J. Hum. Lact..

[29] Almadani M., Vydelingum V., Lawrence J. (2010). Saudi Mothers’ Expected Intentions and Attitudes Toward Breast-Feeding. Infant Child Adolesc. Nutr..

[30] Economou M., Kolokotroni O., Paphiti-Demetriou I., Kouta C., Lambrinou E., Hadjigeorgiou E., Hadjiona V., Middleton N. (2021). The association of breastfeeding self-efficacy with breastfeeding duration and exclusivity: Longitudinal assessment of the pre-dictive validity of the Greek version of the BSES-SF tool. BMC Pregnancy Childbirth.

[31] Mercan Y., Tari Selcuk K. (2021). Association between postpartum depression level, social support level and breastfeeding attitude and breastfeeding self-efficacy in early postpartum women. PLoS ONE.

[32] Ip W.Y., Gao L.L., Choi K.C., Chau J.P., Xiao Y. (2016). The Short Form of the Breastfeeding Self-Efficacy Scale as a Prognostic Factor of Exclusive Breastfeeding among Mandarin-Speaking Chinese Mothers. J. Hum. Lact. Off. J. Int. Lact. Consult. Assoc..

[33] Alyousefi N., Alemam A., Altwaijri D., Alarifi S., Alessa H. (2022). Predictors of Prenatal Breastfeeding Self-Efficacy in Expectant Mothers with Gestational Diabetes Mellitus. Int. J. Environ. Res. Public Health.

[34] Colombo L., Crippa B.L., Consonni D., Bettinelli M.E., Agosti V., Mangino G., Bezze E.N., Mauri P.A., Zanotta L., Roggero P. (2018). Breastfeeding Determinants in Healthy Term Newborns. Nutrients.

[35] Waits A., Guo C.Y., Chien L.Y. (2018). Evaluation of factors contributing to the decline in exclusive breastfeeding at 6 months postpartum: The 2011–2016 National Surveys in Taiwan. Birth.

[36] Bandura A., Pastorelli C., Barbaranelli C., Caprara G.V. (1999). Self-efficacy pathways to childhood depression. J. Pers. Soc. Psychol..

[37] Elgzar W.T., Al-Thubaity D.D., Alshahrani M.A., Essa R.M., Ibrahim H.A. (2023). The Relationship between Maternal Ideation and Exclusive Breastfeeding Practice among Saudi Nursing Mothers: A Cross-Sectional Study. Nutrients.

[38] Gerhardsson E., Nyqvist K.H., Mattsson E., Volgsten H., Hildingsson I., Funkquist E.L. (2014). The Swedish Version of the Breastfeeding Self-Efficacy Scale-Short Form: Reliability and Validity Assessment. J. Hum. Lact. Off. J. Int. Lact. Consult. Assoc..

[39] Tsaras K., Sorokina T., Papathanasiou I.V., Fradelos E.C., Papagiannis D., Koulierakis G. (2021). Breastfeeding Self-efficacy and Related Socio-demographic, Perinatal and Psychological Factors: A Cross-sectional Study Among Postpartum Greek Women. Mater. Socio-Med..

[40] Amin T., Hablas H., Al Qader A.A. (2011). Determinants of initiation and exclusivity of breastfeeding in Al Hassa, Saudi Arabia. Breastfeed. Med. Off. J. Acad. Breastfeed. Med..

[41] Rocha I.S., Lolli L.F., Fujimaki M., Gasparetto A., Rocha NB D. (2018). Influence of maternal confidence on exclusive breastfeeding until six months of age: A systematic review. Influência da autoconfiança materna sobre o aleitamento materno exclusivo aos seis meses de idade: Uma revisão sistemática. Cienc. Saude Coletiva.

[42] Monteiro J.C.D.S., Guimarães C.M.S., Melo L.C.O., Bonelli M.C.P. (2020). Breastfeeding self-efficacy in adult women and its relationship with exclusive maternal breastfeeding. Rev. Lat.-Am. Enferm..

[43] Moraes G.G.W., Christoffel M.M., Toso B.R.G.O., Viera C.S. (2021). Association between duration of exclusive breastfeeding and nursing mothers’ self-efficacy for breastfeeding. Rev. Esc. Enferm. USP.

[44] Titaley C.R., Dibley M.J., Ariawan I., Mu’asyaroh A., Alam A., Damayanti R., Do T.T., Ferguson E., Htet K., Li M. (2021). Determinants of low breastfeeding self-efficacy amongst mothers of children aged less than six months: Results from the BADUTA study in East Java, Indonesia. Int. Breastfeed. J..

[45] Titaley C.R., Loh P.C., Prasetyo S., Ariawan I., Shankar A.H. (2014). Socio-economic factors and use of maternal health services are associated with delayed initiation and non-exclusive breastfeeding in Indonesia: Secondary analysis of Indonesia Demographic and Health Surveys 2002/2003 and 2007. Asia Pac. J. Clin. Nutr..

[46] AlSedra H., AlQurashi A.A. (2022). Exploring the Experience of Breastfeeding Among Working Mothers at Healthcare Facility in Saudi Arabia: A Qualitative Approach. Cureus.

[47] Awoke N., Tekalign T., Lemma T. (2020). Predictors of optimal breastfeeding practices in Worabe town, Silte zone, South Ethiopia. PLoS ONE.

[48] Dodt R.C., Joventino E.S., Aquino P.S., Almeida P.C., Ximenes L.B. (2015). An experimental study of an educational intervention to promote maternal self-efficacy in breastfeeding. Rev. Lat.-Am. Enferm..

[49] Mirghafourvand M., Malakouti J., Mohammad-Alizadeh-Charandabi S., Faridvand F. (2018). Predictors of Breastfeeding Self-efficacy in Iranian Women: A Cross-Sectional Study. Int. J. Womens Health Reprod. Sci..

[50] Hegazi M.A., Allebdi M., Almohammadi M., Alnafie A., Al-Hazmi L., Alyoubi S. (2019). Factors associated with exclusive breastfeeding in relation to knowledge, attitude and practice of breastfeeding mothers in Rabigh community, Western Saudi Arabia. World J. Pediatr. WJP.

